# Behavior of hospitalized severe influenza cases according to the outcome variable in Catalonia, Spain, during the 2017–2018 season

**DOI:** 10.1038/s41598-021-92895-5

**Published:** 2021-06-30

**Authors:** Núria Soldevila, Lesly Acosta, Ana Martínez, Pere Godoy, Núria Torner, Cristina Rius, Mireia Jané, Angela Domínguez, the Surveillance of Hospitalized Cases of Severe Influenza in Catalonia Working Group, M. Alsedà, J. Álvarez, C. Arias, P. J. Balañà, I. Barrabeig, N. Camps, M. Carol, J. Ferràs, G. Ferrús, N. Follia, P. Bach, S. Minguell, I. Parrón, E. Plasència, M. R. Sala-Farré, R. Torra, J. Torres, M. A. Marcos, M. M. Mosquera, A. Vilella, A. Antón, T. Pumarola, M. Campins, D. García, A. Oller Perez-Hita, E. Espejo, N. Freixas, M. Riera Garcia, E. Maraver, D. Mas, R. Pérez, J. Rebull, J. Pou, G. García-Pardo, M. Olona, F. Barcenilla, D. Castellana, G. Navarro-Rubio, L. Force, J. M. Mòdol-Deltell, G. Mena, L. Matas, A. Alvarez, J. M. Torrel

**Affiliations:** 1 CIBER Epidemiología y Salud Pública (CIBERESP), Madrid, Spain; 2 Departament de Medicina, Universitat de Barcelona, C/Casanova, 143, 08036 Barcelona, Spain; 3 Universitat Politècnica de Catalunya-BCNTECH, Barcelona, Spain; 4 Agència de Salut Pública de Catalunya, Generalitat de Catalunya, Barcelona, Spain; 5 Agència de Salut Pública de Barcelona, Barcelona, Spain; 6 Hosptial Clínic de Barcelona, Barcelona, Spain; 7 Hospital Universitari Vall d’Hebrón, Barcelona, Spain; 8 Hospital Josep Trueta, Girona, Spain; 9 Hospital de Terrassa, Terrassa, Spain; 10 Hospital Mútua de Terrassa, Terrassa, Spain; 11 Hospital Altahia de Manresa, Manresa, Spain; 12 Hospital Verge de la Cinta, Tortosa, Spain; 13 Hospital Sant Joan de Déu, Esplugues de Llobregat, Spain; 14 Hospital Joan XXIII, Tarragona, Spain; 15 Hospital Arnau de Vilanova, Lleida, Spain; 16 Consorci Sanitari Parc Taulí, Sabadell, Spain; 17 Hospital de Mataró, Mataró, Spain; 18 Hospital Germans Trias i Pujol, Badalona, Spain; 19 Hospital Universitari de Bellvitge, Barcelona, Spain

**Keywords:** Infectious diseases, Influenza virus, Risk factors

## Abstract

Influenza is an important cause of severe illness and death among patients with underlying medical conditions and in the elderly. The aim of this study was to investigate factors associated with ICU admission and death in patients hospitalized with severe laboratory-confirmed influenza during the 2017–2018 season in Catalonia. An observational epidemiological case-to-case study was carried out. Reported cases of severe laboratory-confirmed influenza requiring hospitalization in 2017–2018 influenza season were included. Mixed-effects regression analysis was used to estimate the factors associated with ICU admission and death. A total of 1306 cases of hospitalized severe influenza cases were included, of whom 175 (13.4%) died and 217 (16.6%) were ICU admitted. Age 65–74 years and ≥ 75 years and having ≥ 2 comorbidities were positively associated with death (aOR 3.19; 95%CI 1.19–8.50, aOR 6.95, 95%CI 2.76–1.80 and aOR 1.99; 95%CI 1.12–3.52, respectively). Neuraminidase inhibitor treatment and pneumonia were negatively associated with death. The 65–74 years and ≥ 75 years age groups were negatively associated with ICU admission (aOR 0.41; 95%CI 0.23–0.74 and aOR 0.30; 95%CI 0.17–0.53, respectively). A factor positively associated with ICU admission was neuraminidase inhibitor treatment. Our results support the need to investigate the worst outcomes of hospitalized severe cases, distinguishing between death and ICU admission.

## Introduction

Influenza affects 10–20% of the unvaccinated population each year and is a leading cause of severe illness and death among patients with underlying medical conditions and in people aged ≥ 65 years^1^. Worldwide, annual epidemics are estimated to result in about 3 to 5 million cases of severe illness and about 290,000 to 650,000 deaths caused by respiratory conditions^2^. Most influenza infections are self-limiting, requiring no healthcare visits, but a proportion of cases present severe complications and require hospitalization and ICU admission.

ICU admission and death have been considered as the worst outcomes for severe cases of influenza and sometimes only a category including those patients requiring ICU admission or who died is considered to identify the factors associated with severity^3,4^. However, because some differences have been found according to the outcome considered^5,6^, it seems reasonable to look at these different outcomes in a separate analysis.

In the 1999–2000 season, a sentinel surveillance program of influenza and other viral diseases based on physicians of primary healthcare centers was initiated in Catalonia, a region in the northeast of Spain.

In the 2010–2011 season, the surveillance of these diseases was improved by adding information about severe hospitalized influenza cases that were laboratory-confirmed provided by sentinel hospitals. In the 2017–2018 season there were 14 public hospitals, 13 attending adult and pediatric patients and one attending only pediatric patients. The hospitals participating in this sentinel system cover a population of 4,950,851, which represents 66.2% of the whole population^7^.

In Catalonia, annual influenza vaccination is recommended to persons aged > 60 years, to pregnant women, and to patients with chronic medical conditions in whom influenza could have severe consequences. Antiviral treatment with neuraminidase inhibitors (NAI) is administered only to hospitalized patients with severe presentation and to those in which individual assessment make it advisable because of high risk for severe complications^8^.

Studies carried out in different geographical areas during the 2017–2018 influenza season showed increased severity in all age groups^9–13^. In Catalonia, according to the healthcare demand, the age-adjusted cumulative incidence rate in the 2017–2018 influenza season was 2295.26 per 100,000 habitants. Influenza B virus accounted for 63% of influenza viruses detected. The predominant lineage was Yamagata whereas the previous season was, with scarce circulation, Victoria lineage; H1N1 influenza A strains were similar to previous seasons, while a proportion of H3N2 influenza A circulating strains were different from the previous season. The difference in circulating influenza viruses, both AH3N2 and B, respect to the strains contained in the vaccine, could be responsible for a greater number of severe cases affecting specially the elderly^14^.

The aim of this study was to investigate factors associated with ICU admission and death in patients hospitalized with severe laboratory-confirmed influenza during the 2017–2018 season.

## Results

**Figure 1:**
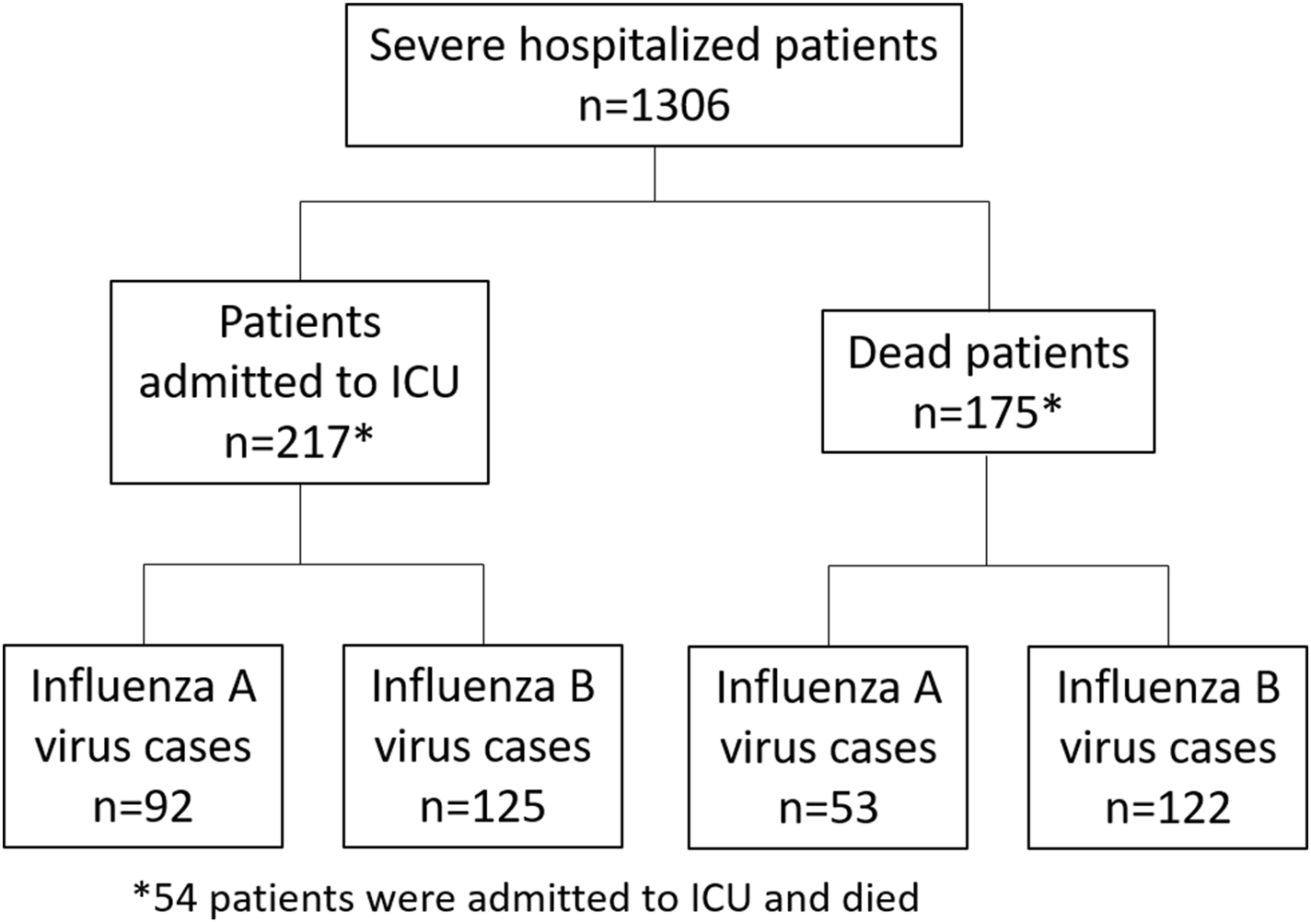
Flow chart of severe hospitalized influenza cases.

A total of 1306 cases of hospitalized severe influenza cases were included, 56.9% (743/1306) were male, the mean of age was 68.0 ± 20.7 and 34.3% (445/1299) had received the influenza vaccine. 518 were influenza A virus cases and 788 were influenza B virus cases; of the influenza A cases that were subtype, 101 were H1 and 80 were H3; no H5 was found. 175 patients (13.4%) died and 217 (16.6%) were admitted to the ICU (Fig. 1).

There was no collinearity or interaction between variables.

Demographic and clinical characteristics of patients who died or survived are shown in Table 1. Age 65–74 and ≥ 75 years, having ≥ 2 comorbidities were positively associated with death and nosocomial infection (aOR 3.19; 95%CI 1.19–8.50, aOR 6.95, 95%CI 2.76–1.80, aOR 1.99; 95%CI 1.12–3.52 and aOR 1.91; 95%CI 1.16–3.17, respectively). NAI treatment, length of stay and pneumonia were negatively associated with death (aOR 0.23; 95%CI 0.14–0.38, aOR 0.51; 95%CI 0.35–0.74 and aOR 0.64; 95%CI 0.43–0.94, respectively). No coinfections with other influenza-type viruses were detected.

**Table 1:** Factors associated with death in hospitalized severe influenza patients.

	Death (N = 175)	No death (N = 1131)	Crude OR (95% CI)	P value	Adjusted OR (95% CI)	P value
**Age**						
< 15 years	0 (0%)	55 (4.9%)	–		–	
15–49 years	6 (3.4%)	131 (11.6%)	Ref		Ref	
50–64 years	22 (12.6%)	240 (21.2%)	2.03 (0.80–5.20)	0.14	2.06 (0.78–5.45)	0.15
65–74 years	27 (15.4%)	240 (21.2%)	2.70 (1.08–6.71)	0.03	3.19 (1.19–8.50)	0.02
≥ 75 years	120 (68.6%)	465 (41.1%)	6.64 (2.85–1.55)	< 0.01	6.95 (2.76–1.80)	< 0.01
**Sex**						
Female	64 (36.6%)	499 (44.1%)	Ref		Ref	
Male	111 (63.4%)	632 (55.9%)	1.39 (0.99–1.93)	0.05	1.38 (0.97–1.96)	0.07
**Size of municipality**						
≤ 10.000 inhabitants	32 (18.3%)	135 (11.9%)	1.54 (0.97–2.42)	0.07	1.13 (0.70–1.84)	0.61
> 10.000 inhabitants	143 (81.7%)	996 (88.1%)	Ref		Ref	
**Comorbidities**						
1	55 (31.4%)	354 (31.3%)	3.60 (1.88–8.86)	< 0.01	1.66 (0.90–3.07)	0.10
≥ 2	108 (61.7%)	515 (45.5%)	4.82 (2.60–8.93)	< 0.01	1.99 (1.12–3.52)	0.02
No	12 (6.9%)	262 (23.2%)	Ref		Ref	
**COPD**						
Yes	53 (30.3%)	287 (25.4%)	1.32 (0.93–1.89)	0.12	0.92 (0.62–1.36)	0.67
No	122 (69.7%)	844 (74.6%)	Ref		Ref	
**Morbid obesity**						
Yes	11 (6.3%)	81 (7.2%)	0.86 (0.44–1.67)	0.66	0.90 (0.46–1.76)	0.76
No	164 (93.7%)	1050 (92.8%)	Ref		Ref	
**Diabetes**						
Yes	48 (27.4%)	280 (24.8%)	1.15 (0.80–1.65)	0.46	0.90 (0.61–1.33)	0.61
No	127 (72.6%)	851 (75.2%)	Ref		Ref	
**Chronic renal disease**						
Yes	49 (28.0%)	173 (15.3%)	2.15 (1.48–3.12)	< 0.01	1.34 (0.89–2.03)	0.16
No	126 (72.0%)	958 (84.7%)	Ref		Ref	
**Immune deficiency**						
Yes	36 (20.6%)	172 (15.2%)	1.38 (0.92–2.07)	0.12	1.40 (0.92–2.12)	0.12
No	139 (79.4%)	959 (84.8%)	Ref		Ref	
**Chronic cardiovascular disease**						
Yes	98 (56.0%)	397 (35.1%)	2.43 (1.75–3.37)	< 0.01	1.40 (0.97–2.03)	0.07
No	77 (44.0%)	734 (64.9%)	Ref		Ref	
**Chronic liver disease**						
Yes	19 (10.9%)	57 (5.0%)	2.16 (1.24–3.77)	< 0.01	1.97 (1.09–3.57)	0.02
No	156 (89.1%)	1074 (95.0%)	Ref		Ref	
**Other comorbidities**^**a**^						
Yes	35 (20.0%)	138 (12.2%)	1.62 (1.07–2.47)	0.02	1.40 (0.90–2.18)	0.13
No	140 (80.0%)	993 (87.8%)	Ref		Ref	
**Pregnancy**						
Yes	0 (0%)	7 (0.6%)	–	**–**	–	–
No	175 (100%)	1124 (99.4%)	Ref		Ref	
**Length of stay**						
0–6 days	79 (45.1%)	428 (37.8%)	Ref		Ref	
≥ 7 days	96 (54.9%)	703 (62.2%)	0.70 (0.50–0.97)	0.03	0.51 (0.35–0.74)	< 0.01
**Type of virus**						
A	53 (30.3%)	465 (41.1%)	0.63 (0.45–0.89)	0.01	0.82 (0.57–1.18)	0.28
B	122 (69.7%)	666 (58.9%)	Ref		Ref	
**Seasonal vaccine**						
Yes	71 (41.5%)	374 (33.2%)	1.58 (1.13–2.22)	< 0.01	1.02 (0.71–1.47)	0.90
No	100 (58.5%)	754 (66.8%)	Ref		Ref	
**NAI treatment**						
Yes	141 (80.6%)	1052 (93.0%)	0.34 (0.22–0.53)	< 0.01	0.23 (0.14–0.38)	< 0.01
No	34 (19.4%)	79 (7.0%)	Ref		Ref	
**NAI treatment**						
≤ 48 h symptom onset	62 (36.3%)	413 (37.4%)	0.40 (0.24–0.65)	< 0.01	0.27 (0.16–0.47)	< 0.01
> 48 h symptom onset	75 (43.9%)	611 (55.4%)	0.30 (0.19–0.49)	< 0.01	0.22 (0.13–0.37)	< 0.01
No	34 (19.9%)	79 (7.2%)	Ref		Ref	
**Pneumonia**						
Yes	105 (60.7%)	726 (64.2%)	0.61 (0.42–0.89)	0.01	0.64 (0.43–0.94)	0.02
No	68 (39.3%)	404 (35.8%)	Ref		Ref	
**Nosocomial infection**						
Yes	31 (17.8%)	111 (9.8%)	2.25 (1.40–3.63)	< 0.01	1.91 (1.16–3.17)	0.01
No	143 (82.2%)	1020 (90.2%)	Ref		Ref	

COPD: chronic obstructive pulmonary disease, NAI: neuraminidase inhibitors.

^a^Hemoglobinopathy, severe neuromuscular disease or cognitive dysfunction.

**Figure 2:**
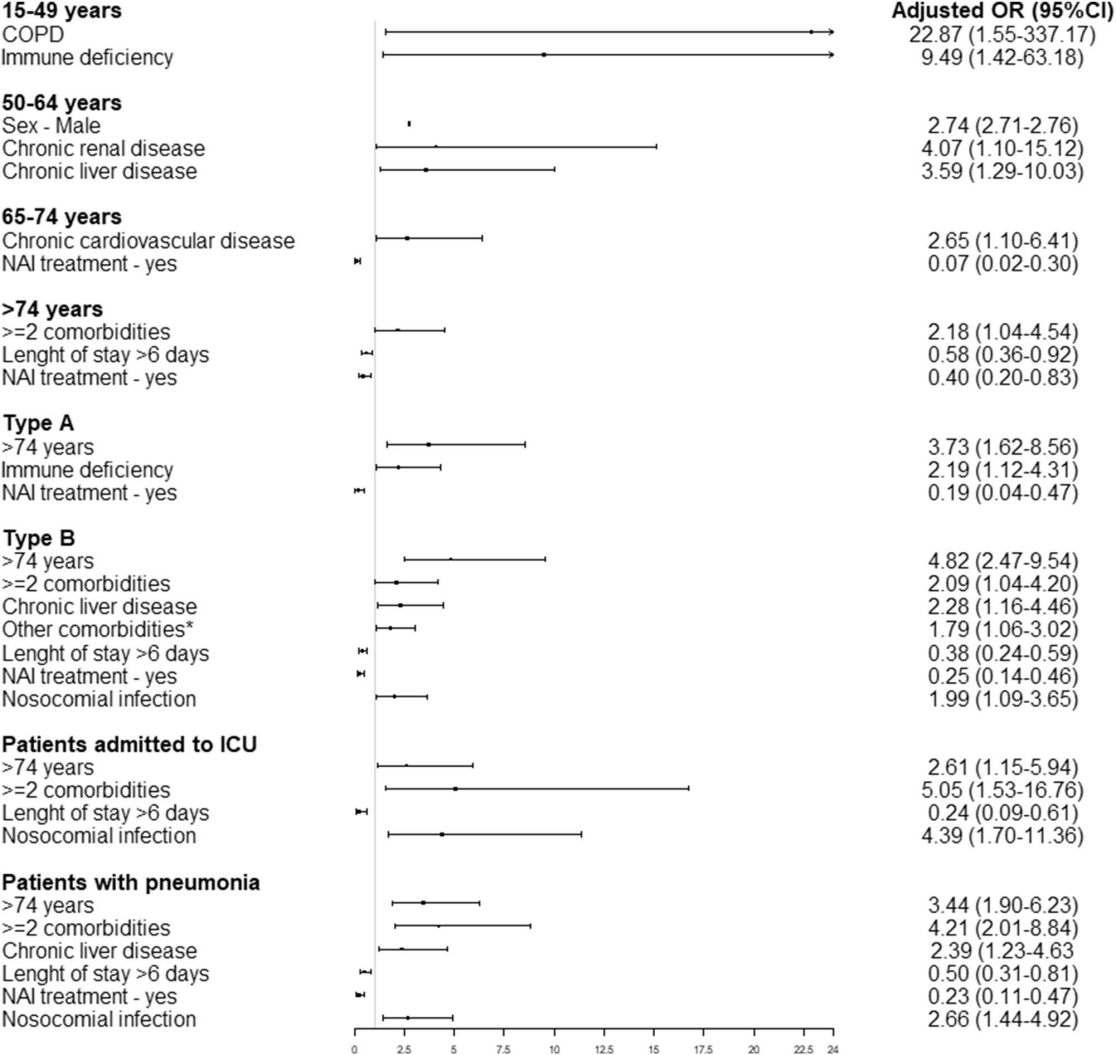
Factors associated with death in hospitalized severe influenza patients according to age group, influenza type, patients admitted to ICU and patients with pneumonia. *Hemoglobinopathy, severe neuromuscular disease or cognitive dysfunction. COPD: chronic obstructive pulmonary disease, NAI: neuraminidase inhibitors.

Factors associated with death according to patient groups are summarized in Fig. 2. The factors with the highest positive associations with death were chronic renal disease and chronic liver disease in the 50–64 years age group, chronic cardiovascular disease in the 65–74 years age group and having ≥ 2 comorbidities in the ≥ 75 years age group. Male sex was associated with death only in the 50–64 years age group.

In influenza A virus cases, the factors positively associated with death were age ≥ 75 years and immune deficiency, while NAI treatment was negatively associated with death.

In influenza B virus cases, factors positively associated with death were age ≥ 75 years, chronic liver disease, having ≥ 2 comorbidities and nosocomial infection. Length of stay and NAI treatment was negatively associated with death.

In patients admitted to the ICU, the proportion of death was 24.9% (54/217) and factors positively associated with death were age ≥ 75 years, having ≥ 2 comorbidities and nosocomial infection.

In patients who presented pneumonia, the proportion of death was 12.6% (105/831) and factors positively associated with death were ≥ 2 chronic medical conditions, age ≥ 75 years, chronic liver disease and nosocomial infection. Length of stay and NAI treatment was negatively associated with death.

The demographic and clinical characteristics of patients admitted to the ICU or not are shown in Table 2. Age < 15 years, 65–74 years or ≥ 75 years were negatively associated with ICU admission (aOR 0.29; 95%CI 0.11–0.76, aOR 0.41; 95%CI 0.23–0.74 and aOR 0.30; 95%CI 0.17–0.53, respectively) as was pneumonia (aOR 0.61; 95%CI 0.41–0.89). NAI treatment and length of stay was positively associated with ICU admission (aOR 2.12; 95%CI 1.19–4.13 and aOR 6.54; 95%CI 4.17–10.24, respectively).

**Table 2:** Factors associated with ICU admission in hospitalized severe influenza patients.

	ICU admission (N = 217)	No ICU admission (N = 1089)	Crude OR (95% CI)	P value	Adjusted OR (95% CI)	P value
**Age**						
< 15 years	6 (2.8%)	49 (4.5%)	0.28 (0.11–0.74)	0.01	0.29 (0.11–0.76)	0.01
15–49 years	34 (15.7%)	103 (9.5%)	Ref		Ref	
50–64 years	79 (36.4%)	183 (16.8%)	1.30 (0.79–2.13)	0.30	1.17 (0.71–1.94)	0.53
65–74 years	37 (17.1%)	230 (21.1%)	0.50 (0.29–0.86)	0.01	0.41 (0.23–0.74)	< 0.01
≥ 75 years	61 (28.1%)	524 (48.1%)	0.39 (0.24–0.64)	< 0.01	0.30 (0.17–0.53)	< 0.01
**Sex**						
Female	81 (37.3%)	482 (44.3%)	Ref		Ref	
Male	136 (62.7%)	607 (55.7%)	1.36 (0.99–1.86)	0.05	1.31 (0.95–1.81)	0.10
**Size of municipality**						
≤ 10.000 inhabitants	41 (18.9%)	126 (11.6%)	1.33 (0.86–2.07)	0.20	1.48 (0.94–2.33)	0.09
> 10.000 inhabitants	176 (81.1%)	963 (88.4%)	Ref		Ref	
**Comorbidities**						
1	70 (32.3%)	339 (31.1%)	1.06 (0.70–1.63)	0.77	1.14 (0.72–1.76)	0.60
≥ 2	101 (46.5%)	552 (47.9%)	0.97 (0.65–1.45)	0.90	1.08 (0.76–1.84)	0.46
No	46 (21.2%)	228 (20.9%)	Ref		Ref	
**COPD**						
Yes	60 (27.6%)	280 (25.7%)	1.09 (0.77–1.54)	0.63	1.11 (0.76–1.60)	0.60
No	157 (72.4%)	809 (74.3%)	Ref		Ref	
**Morbid obesity**						
Yes	23 (10.6%)	69 (6.3%)	1.87 (1.08–3.22)	0.02	1.66 (0.95–2.89)	0.07
No	194 (89.4%)	1020 (93.7%)	Ref		Ref	
**Diabetes**						
Yes	59 (27.2%)	269 (24.7%)	1.15 (0.81–1.62)	0.43	1.24 (0.87–1.78)	0.23
No	158 (72.8%)	820 (75.3%)	Ref		Ref	
**Chronic renal disease**						
Yes	28 (12.9%)	194 (17.8%)	0.65 (0.42–1.01)	0.06	0.78 (0.48–1.25)	0.30
No	189 (87.1%)	895 (82.2%)	Ref		Ref	
**Immune deficiency**						
Yes	33 (15.2%)	175 (16.1%)	0.91 (0.60–1.38)	0.65	0.86 (0.56–1.33)	0.50
No	184 (84.8%)	914 (83.9%)	Ref		Ref	
**Chronic cardiovascular disease**						
Yes	79 (36.4%)	416 (38.2%)	0.98 (0.71–1.34)	0.90	1.12 (0.78–1.59)	0.54
No	138 (63.6%)	673 (61.8%)	Ref		Ref	
**Chronic liver disease**						
Yes	24 (11.1%)	52 (4.8%)	2.01 (1.18–3.42)	0.01	1.75 (1.01–3.06)	0.05
No	193 (88.9%)	1037 (95.2%)	Ref		Ref	
**Other comorbidities**^**a**^						
Yes	28 (12.9%)	145 (13.3%)	0.82 (0.52–1.29)	0.39	0.92 (0.58–1.47)	0.74
No	189 (87.1%)	944 (86.7%)	Ref		Ref	
**Pregnancy**						
Yes	2 (0.9%)	5 (0.5%)	1.84 (0.33–10.31)	0.49	1.74 (0.31–9.80)	0.53
No	215 (99.1%)	1084 (99.5%)	Ref		Ref	
**Length of stay**						
0–6 days	26 (12.0%)	481 (44.2%)	Ref		Ref	
≥ 7 days	191 (88.0%)	608 (55.8%)	6.68 (4.30–10.38)	< 0.01	6.54 (4.17–10.24)	< 0.01
**Type of virus**						
A	92 (42.4%)	426 (39.1%)	1.20 (0.88–1.64)	0.24	1.14 (0.83–1.57)	0.41
B	125 (57.6%)	663 (60.9%)	Ref		Ref	
**Seasonal vaccine**						
Yes	52 (24.1%)	393 (36.3%)	0.62 (0.44–0.88)	< 0.01	0.80 (0.55–1.16)	0.24
No	164 (75.9%)	690 (63.7%)	Ref		Ref	
**NAI treatment**						
Yes	202 (93.1%)	991 (91.0%)	1.90 (1.06–3.41)	0.03	2.12 (1.19–4.13)	0.01
No	15 (6.9%)	98 (9.0%)	Ref		Ref	
**NAI treatment**						
≤ 48 h symptom onset	60 (28.8%)	415 (38.9%)	1.55 (0.83–2.92)	0.17	1.83 (0.94–3.57)	0.07
> 48 h symptom onset	133 (63.9%)	553 (51.9%)	2.02 (1.11–3.66)	0.02	2.38 (1.26–4.49)	0.01
No	15 (7.2%)	98 (9.2%)	Ref		Ref	
**Pneumonia**						
Yes	151 (69.6%)	680 (62.6%)	0.65 (0.44–0.94)	0.02	0.61 (0.41–0.89)	0.01
No	66 (30.4%)	406 (37.4%)	Ref		Ref	
**Nosocomial infection**						
Yes	22 (10.2%)	120 (11.0%)	1.16 (0.68–1.99)	0.58	1.08 (0.62–1.88)	0.79
No	194 /89.8%)	969 (89.0%)	Ref		Ref	

COPD: chronic obstructive pulmonary disease, NAI: neuraminidase inhibitors.

^a^Hemoglobinopathy, severe neuromuscular disease or cognitive dysfunction.

**Figure 3:**
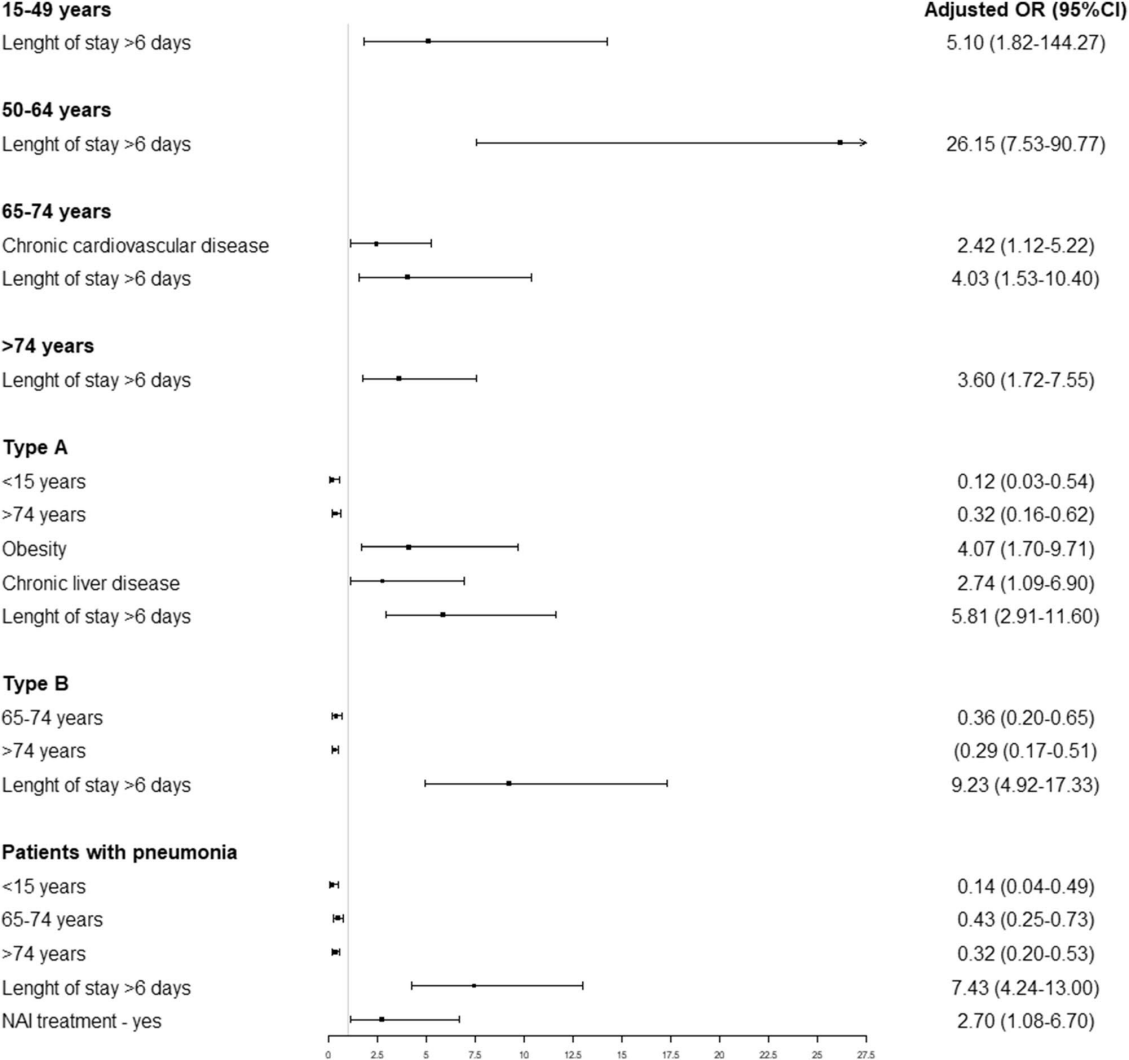
Factors associated with ICU admission in hospitalized severe influenza patients according to age group, influenza type and patients with pneumonia. NAI: neuraminidase inhibitors.

Factors associated with ICU admission according to patient subgroup are summarized in Fig. 3. Chronic vascular disease was positively associated with ICU admission only in the 65–74 years age group.

In influenza A virus cases, factors associated positively with ICU admission were morbid obesity and chronic liver disease. Factors negatively associated with ICU admission were the < 15 years and ≥ 75 years age groups.

In influenza B virus cases, factors negatively associated were the 65–74 years and ≥ 75 years age groups.

In patients with pneumonia, length of stay and NAI treatment was associated positively with ICU admission and factors negatively associated with ICU admission were the < 15 years, 65–74 years and ≥ 75 years age groups.

## Discussion

The results of this study show that the proportion of death in hospitalized severe influenza cases was 13.4%, lower than the 18.8% found by Liu et al.^15^ in the 2016–2018 season in severe patients in Taiwan, but higher than the 9.2% found by Kolosova et al.^16^ in Russia in hospitalized severe patients in the 2017–2018 season, the 6.5% found in all hospitalized patients in China in the same 2017–2018 season^10^, and the 5.5% found by Natheglian et al. in hospitalized severe patients during 2015–2018 in Iran^12^.

The risk of death increased in the 65–74 years and ≥ 75 years age groups, coinciding with the findings of most studies reviewed^12,17–23^, although some authors found no differences in mortality according to age^24^.

Among the factors investigated, COPD was associated with death in the 15–49 years age group, coinciding with the results found by Walker et al. in a study carried out in New Zealand between 2012 and 2015 where the outcome did not distinguish between death and ICU admission^25^. Immunodeficiency was associated with death in the 15–49 years age group in influenza A and B virus cases and in all age groups in influenza A virus cases, and although we do not have the precise information, the association in the first age group was probably was associated with HIV infection, which has been described as a factor of severity for influenza^1^, especially for influenza A cases^26,27^. Czaja et al.^28^ in the United States during the 2010–2011 to 2014–2015 seasons found that, in hospitalized influenza cases, immunosuppression was also associated with death or transfer to a hospice.

Male sex was only associated with death in the 50–64 years age group. Some authors have found male sex was associated with a higher risk of death^12^ but, coinciding with our results, Lina et al. in the United States in the 2017–2018 season found that the death was more frequent in males than females in the 50–64 years age group^29^. Zou et al.^13^ in a study carried out in influenza-related cases in China in the 2017–2018 season found an association between male sex and death in patients with influenza-related pneumonia, but we did not.

As reported by other authors^10,25,26,30^, we found that chronic renal disease was associated with death, but only in the 50–64 years age group.

Nosocomial infection was associated with death. A study carried out in France in 2016–2017 season also found that nosocomial influenza was associated with mortality^31^.

Having received seasonal influenza vaccine was associated with a higher risk of death in the crude analysis but not in the adjusted analysis. This finding could be explained because vaccinated could attend to the hospital more frequently than do unvaccinated subjects as suggested by Casado et al.^32^ in a study carried out in older adults in 2013–2014 and 2014–2015 season. However, residual confounding by comorbid conditions could not be ruled out as suggested by other authors^33^ because patients with comorbidities (79% in our study) are more likely to access healthcare resources and subsequently have more opportunities to receive the influenza vaccine. On the other hand, as suggested by Gutiérrez-González et al.^17^ because only hospitalized severe cases are included in the study, the protection for fatal episodes might be underestimated.

We should also highlight that influenza B virus was the most prevalent in the 2017–2018 influenza season in Catalonia and that the effectiveness of vaccination was only 14% (95%CI 0–47) against influenza B virus cases because the circulating lineage Yamagata was not included in vaccines used^14^.

NAI treatment was a preventive factor against death in all patients considered globally, in the age groups studied, and in influenza A and B virus cases, as other authors have reported^20^. However, some authors^34,35^ found no association between NAI treatment and death. Groenveld et al., in a study carried out in the Netherlands in the 2013–2016 seasons, found that NAI did not avoid death during the hospital stay but did avoid a composite endpoint including 30-day mortality and ICU admission^36^.

In patients with pneumonia, we also found that NAI treatment was a protective factor against death, without differences according to the influenza virus type. Chen et al., in a study carried out in influenza A-related pneumonia cases in China from 2013 to 2018, found that early NAI treatment was protective against death^37^ and that protection was higher in influenza B-related pneumonia cases^38^. Zou et al. found no association between NAI treatment and death and proposed that a double dose of oseltamivir should be administered in patients with pneumonia^13^.

The proportion of hospitalized severe influenza cases admitted to the ICU was 16.6% (17.8% for influenza A cases and 15.9% for influenza B cases), figures close to the 15.4% (23% for influenza A cases and 12.7% for influenza B cases) obtained by Stahl et al.^39^ in Germany in the same influenza season, and the 13.4% for influenza A cases and 17.8% for influenza B cases obtained by Assaf-Casals et al.^24^ in Lebanon during 2008–2016. Other authors studying severe patients have found higher figures^29^, but the differences in criteria for hospitalization might, at least in part, explain the differences.

The highest proportion of ICU admission was found in the 50–64 years age group, and declined thereafter, with the lowest proportion (10.4%) in the ≥ 75 years age group. This finding was also observed by Oliva et al. in a study carried out in hospitalized severe cases in Spain in the 2010–2011 to 2015–2016 seasons, who found that the 15–64 years age group had the highest proportion of ICU admission^19^. Beumer et al. found differences in ICU admission according to age: the highest proportion was in the 50–65 years age group and the proportion fell in patients aged ≥ 65 years^30^. A French study by Pivette et al. in 2012–2017 also found a reduction in ICU admissions in elderly patients with influenza^40^.

Mazagatos et al.^41^ in a Spanish study carried out in the 2010–2011 to 2015–2016 seasons found that 38.9% of hospitalized pregnant women who presented severe influenza were admitted to the ICU, but only 7 pregnant women were included in our study with 2 (29%) requiring ICU admission, and therefore this finding cannot be evaluated. In a systematic review by Mertz et al.^23^ pregnancy as a risk factor was not well studied because of the 56 studies included in the revision only one study having data on this. Coinciding with Walker et al.^25^, ICU admission was associated with cardiovascular disease in patients aged 65–74 years. Our results and those of Beumer et al. in a study carried out in 2015–2016 in the Netherlands also show a positive association between cardiovascular disease and the risk of ICU admission^30^.

We found that morbid obesity was not associated with ICU admission or death, these findings were also observed by Atamna el al. in the study carried out in Israel in 2017–2018 season^42^. In contrast with the results found by Segaloff et al. in a study carried out in Michigan in seasons 2012–2012 and 2012–2013 in adults^43^. Chronic vascular disease in the 65–74 years age group and morbid obesity in influenza A cases were associated with ICU admission, in contrast to Beumer et al., who found no association with obesity^30^.

ICU admission was required in 18.1% of patients with influenza-related pneumonia, lower than the 22.4% obtained in Chinese studies by Chen et al. during 2013–2019^44^ and Zou et al. in the 2027–2018 season^13^. As in all patients considered globally, we found that in patients with pneumonia ICU admission was negatively associated with older age.

When death was the considered outcome, NAI treatment, whether administered in the first 48 h after symptom onset or > 48 h after symptom onset had a protective effect. However, NAI treatment administered > 48 h after symptom onset was associated with ICU admission, a result found in a previous study of including six influenza seasons^45^; this might be because the severity of patients candidates for ICU admission are treated with NAI if they had not been treated before.

Influenza causes a range of outcomes in children, including severe disease, ICU admission and death^3,9^. The case fatality rates of influenza in children range between 0 and 4.9%^6^. In the present study, no deaths were registered in children and no differences in the severity, measured as ICU admission, was found between influenza A and B viruses, coinciding with the findings of other authors^46,47^.

This study, like all observational studies, has strengths and limitations. The main strength is that there are few studies comparing specific outcomes of severity (death on the one hand and ICU admission on the other) and, therefore, our results may help to identify differences between the two outcomes. Another strength is that all cases were laboratory-confirmed and, therefore, the distribution of independent variables corresponded to real severe influenza cases.

The first limitation is that hospitals participated voluntarily, which could lead to selection bias. However, because the hospitals participating in the surveillance system of severe cases cover more than 66% of the Catalan population, the proportion of patients living in municipalities of ≤ 10,000 inhabitants is 12.8%, similar to the total of Catalonia, where the proportion of the population living in municipalities of ≤ 10,000 inhabitants is 18.5% and we used a mixed-effect logistic regression model with hospitals as a random intercept, we believe the results maybe extensible to hospitalized severe cases in Catalonia. Another limitation is that we only studied one influenza season, when the inclusion of several seasons is recommended due to differences in circulating types and subtypes from one season to another. However, because the 2017–2018 influenza season showed high severity, determining the outcomes of severity in this specific season may be interest. A third limitation is that the small number of pregnant women and children did not permit any conclusions about these two key risk groups for influenza severity.

The final adjusted model was constructed taking into account the propensity score built with variables that included the most important comorbidities, and it seems unlikely that the results suffered a large bias. Nevertheless, some residual confounding cannot be ruled out.

In conclusion, our results support the need to investigate the worst outcomes in hospitalized severe cases of influenza, distinguishing between death and ICU admission. We found that older age was a risk factor for death but was negatively associated with ICU admission. By contrast, while NAI treatment both before and after 48 h of symptom onset prevented death, NAI treatment ≥ 48 h after symptom onset was more frequent in patients admitted to the ICU, probably reflecting medical attitudes to patients requiring ICU admission.

## Material and methods

### Study design

An observational, epidemiological case-to-case study was carried out in patients hospitalized due to severe acute influenza infection. A severe case of laboratory-confirmed influenza virus infection was defined as a case requiring hospitalization due to pneumonia, septic shock, multi-organ failure, acute respiratory distress, death or any other severe condition including ICU admission, or who developed these clinical signs during hospitalization for other reasons^8^. The diagnosis was confirmed by RT-PCR and/or culture on nasopharyngeal swab samples.

Respiratory tract samples were processed at each hospital laboratory within 24 h of receipt. A 300 μl aliquot was taken for total nucleic acid extraction and eluted in 25 μl of RNase-free elution buffer using the automatic QIAsymphony system (Qiagen, Hilden, Germany) according to the manufacturer’s instructions. Subsequently, two specific one-step multiplex real-time PCR using Stratagene Mx3000P QPCR Systems (Agilent Technologies, Santa Clara, CA, USA) were carried out for typing A/B influenza virus (sensitivity was 10 and 103 copies/μl, respectively) and subtyping influenza A virus (sensitivity was 102, 103 and 10 copies/μl for H1, H3 and H5 RNA, respectively)^48^. For H1 positive detections to identify H1N1pdm09, specific in-house primers were used.

### Data collected

Reported cases of severe laboratory-confirmed influenza requiring hospitalization in the 2017–2018 influenza season were included.

For each reported case, trained interviewer collected the following variables: demographic data (age, sex, size of municipally), medical conditions (COPD, morbid obesity [BMI > 40 kg/m^2^ to classify obesity for adults and for children obesity is defined as a BMI at or above the 95th percentile for children and teens of the same age and sex], diabetes, chronic renal disease, immune deficiency [HIV infection or other], chronic cardiovascular disease, chronic liver disease, hemoglobinopathy, severe neuromuscular disease or cognitive dysfunction), and clinical data (date of symptom onset, type of virus [A or B], pneumonia, seasonal influenza vaccination status, antiviral treatment with NAI and its date, ICU admission and in-hospital mortality). Cases were considered vaccinated if they had received a dose of influenza vaccine ≥ 14 days before symptom onset. The primary source of information was the medical record.

### Statistical analysis

We assessed the associations between death or UCI admission and the independent variables (sociodemographic variables, virus type and clinical characteristics). Possible interactions between independent variables were analyzed by logistic regression. Independent variables were checked for collinearity using the variance inflation factor.

Crude and adjusted odds ratio (OR) and their corresponding 95% confidence intervals (CI) were constructed using a mixed-effects logistic regression model with the variable hospital as a random intercept. Adjusted OR (aOR) were calculated using propensity scores, which were estimated by logistic regression with age, sex, comorbidities, pneumonia, seasonal influenza vaccination, type of virus and NAI treatment as independent variables. The propensity score was used as a continuous covariate in the mixed-effects logistic regression model.

The analysis was performed using the SPSS v.25 statistical package and R v3.5.0 statistical software (http://cran.r-project.org).

### Ethical considerations

Data used in the analysis were collected under routine public health surveillance activities as part of the legislated mandate of the Health Department of Catalonia, the competent authority for the surveillance of communicable diseases, which is officially authorized to receive, treat and temporally store personal data on cases of infectious diseases^49^. All data were fully anonymized. All study activities formed part of public surveillance and were thus exempt from institutional board review of Health Department of Government of Catalonia and did not require informed consent.

## Data Availability

The data are available from the corresponding author on reasonable request.
