# Author Correction: Behavior of hospitalized severe influenza cases according to the outcome variable in Catalonia, Spain, during the 2017–2018 season

**DOI:** 10.1038/s41598-021-99937-y

**Published:** 2021-10-12

**Authors:** Núria Soldevila, Lesly Acosta, Ana Martínez, Pere Godoy, Núria Torner, Cristina Rius, Mireia Jané, Angela Domínguez, the Surveillance of Hospitalized Cases of Severe Influenza in Catalonia Working Group, M. Alsedà, J. Álvarez, C. Arias, P. J. Balañà, I. Barrabeig, N. Camps, M. Carol, J. Ferràs, G. Ferrús, N. Follia, P. Bach, S. Minguell, I. Parrón, E. Plasència, M. R. Sala-Farré, R. Torra, J. Torres, M. A. Marcos, M. M. Mosquera, A. Vilella, A. Antón, T. Pumarola, M. Campins, D. García, A. Oller Perez-Hita, E. Espejo, N. Freixas, M. Riera Garcia, E. Maraver, D. Mas, R. Pérez, J. Rebull, J. Pou, G. García-Pardo, M. Olona, F. Barcenilla, D. Castellana, G. Navarro-Rubio, L. Force, J. M. Mòdol-Deltell, G. Mena, L. Matas, A. Alvarez, J. M. Torrel

**Affiliations:** 1 CIBER Epidemiología y Salud Pública (CIBERESP), Madrid, Spain; 2 Departament de Medicina, Universitat de Barcelona, C/Casanova, 143, 08036 Barcelona, Spain; 3 Universitat Politècnica de Catalunya-BCNTECH, Barcelona, Spain; 4 Agència de Salut Pública de Catalunya, Generalitat de Catalunya, Barcelona, Spain; 5 Agència de Salut Pública de Barcelona, Barcelona, Spain; 6 Hosptial Clínic de Barcelona, Barcelona, Spain; 7 Hospital Universitari Vall d’Hebrón, Barcelona, Spain; 8 Hospital Josep Trueta, Girona, Spain; 9 Hospital de Terrassa, Terrassa, Spain; 10 Hospital Mútua de Terrassa, Terrassa, Spain; 11 Hospital Altahia de Manresa, Manresa, Spain; 12 Hospital Verge de la Cinta, Tortosa, Spain; 13 Hospital Sant Joan de Déu, Esplugues de Llobregat, Spain; 14 Hospital Joan XXIII, Tarragona, Spain; 15 Hospital Arnau de Vilanova, Lleida, Spain; 16 Consorci Sanitari Parc Taulí, Sabadell, Spain; 17 Hospital de Mataró, Mataró, Spain; 18 Hospital Germans Trias i Pujol, Badalona, Spain; 19 Hospital Universitari de Bellvitge, Barcelona, Spain

Correction to: *Scientific Reports* 10.1038/s41598-021-92895-5, published online 30 June 2021

G. Mena and L. Matas were omitted from the Surveillance of Hospitalized Cases of Severe Influenza in Catalonia Working Group in the original version of this Article.

The original Article has been corrected.

